# Supplementary material for: The Mimivirus L375 Nudix enzyme hydrolyzes the 5’ mRNA cap
Source: PLoS One. 2021 Sep 28;16(9):e0245820. doi: 10.1371/journal.pone.0245820 (PMC8478210; doi:10.1371/journal.pone.0245820)
Supplement: S1 Raw images — (PDF) [file pone.0245820.s001.pdf]

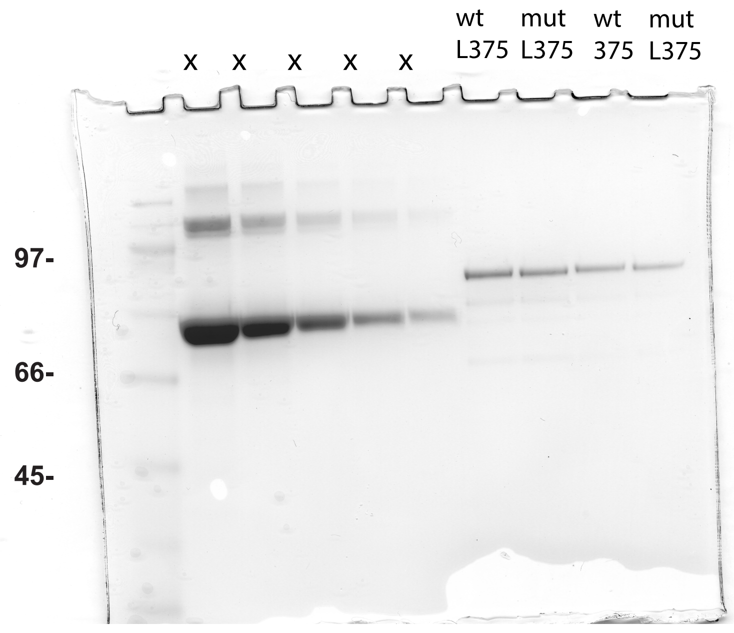

The above figure was used for Fig 2A and 3A. The image is of a Coomassie blue stained gel.

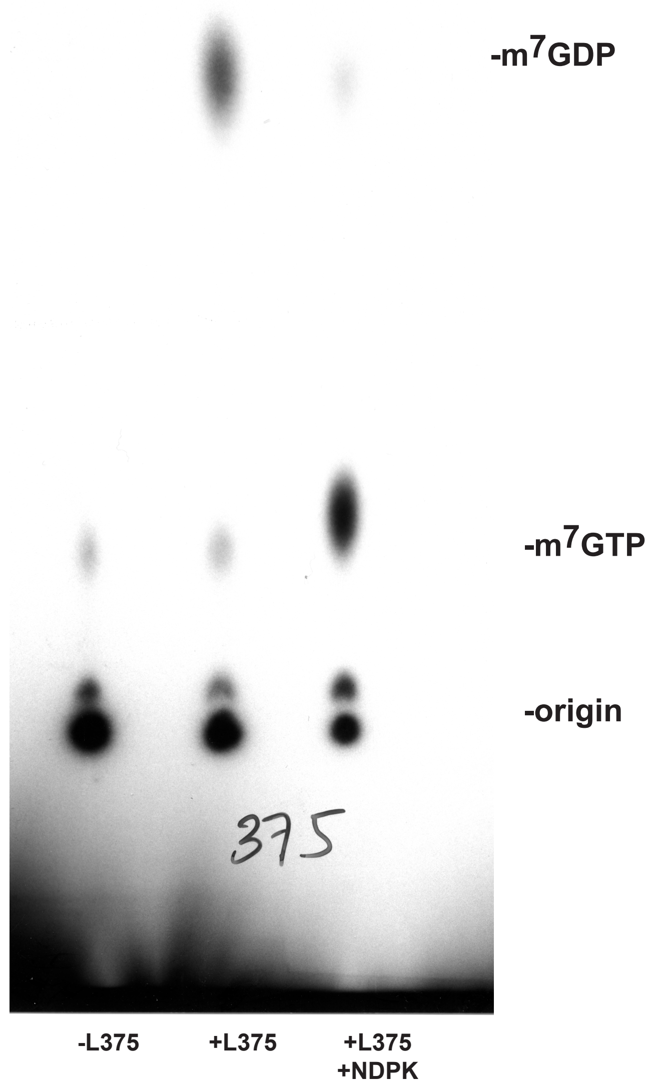

The above figure was used for Fig 2B. This is an X-ray film exposure of a TLC plate.

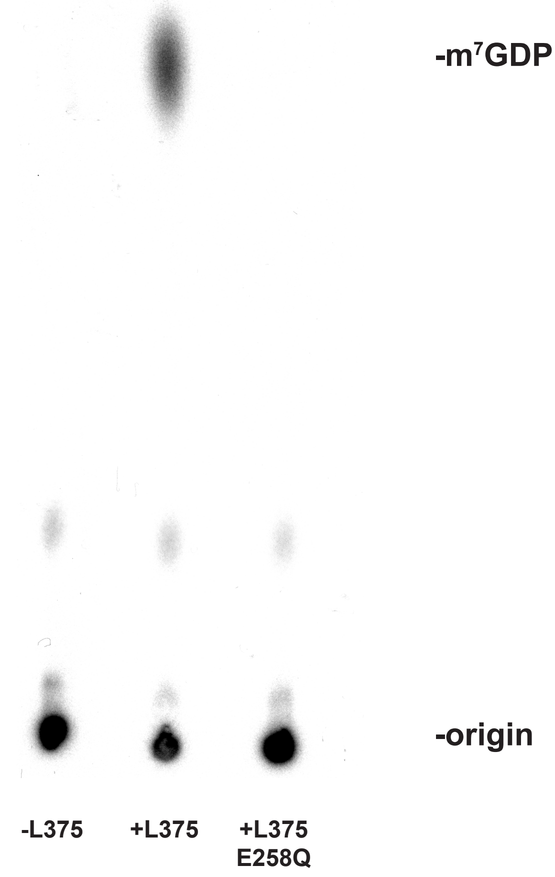

The above figure was used for Fig 3B. This is an X-ray film exposure of a TLC plate.

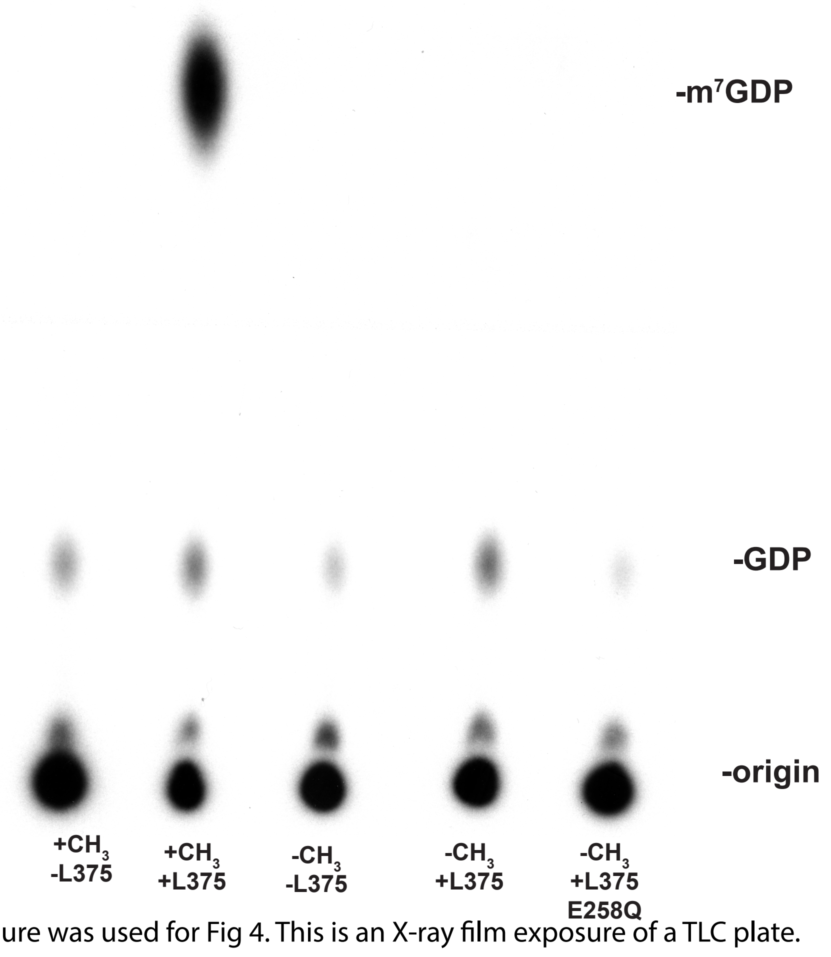

The above figure was used for Fig 4. This is an X-ray film exposure of a TLC plate.
